# Vectorizing Pro-Insecticide: Influence of Linker Length on Insecticidal Activity and Phloem Mobility of New Tralopyril Derivatives

**DOI:** 10.3390/molecules26154570

**Published:** 2021-07-28

**Authors:** Tian Xing Li, Yao Chen, Hui Fang Liu, Chi Yu Ma, Wen Yang

**Affiliations:** 1 College of Tea Science, Guizhou University, Guiyang 550025, China; 18586822040@163.com; 2 Guizhou Tea Research Institute, Guizhou Academy of Agricultural Sciences, Guiyang 550006, China; cheny0221@163.com (Y.C.); 18300865026@163.com (H.F.L.); chiyuma81@163.com (C.Y.M.)

**Keywords:** insecticide conjugate, glutamic acid, tralopyril, chlorfenapyr, phloem mobility

## Abstract

To improve the proinsecticidal activity and phloem mobility of amino acid–tralopyril conjugates further, nine conjugates were designed and synthesized by introducing glutamic acid to tralopyril, and the length of the linker between glutamic acid and tralopyril ranged from 2 atoms to 10 atoms. The results of insecticidal activity against the third-instar larvae of *P. xylostella* showed that conjugates **42**, **43**, **44**,and **45** (straight-chain containing 2–5 atoms) exhibited good insecticidal activity, and their LC_50_ values were 0.2397 ± 0.0366, 0.4413 ± 0.0647, 0.4400 ± 0.0624, and 0.4602 ± 0.0655 mM, respectively. The concentrations of conjugates **43**–**45** were higher than that of conjugate **42** in the phloem sap at 2 h, and conjugate **43** showed the highest concentration. The introduction of glutamic acid can improve phloem mobility. The in vivo metabolism of conjugates **42** and **43** was investigated in *P. xylostella*, and the parent compound tralopyril was detected at concentrations of 0.5950 and 0.3172 nmol/kg, respectively. According to the above results, conjugates **42** and **43** were potential phloem mobile pro-insecticide candidates.

## 1. Introduction

The phloem systemicity of pesticides is an important characteristic for the expression of their biological activities [1,2]. Insecticides with phloem mobility are preferred for controlling pests hidden on unpredictable nonexposed plant parts, such as growing tips, roots, inside leaf deformations, and galls [3]. However, few existing insecticides have good phloem mobility. Currently, only spirotetramat is an ambimobile systemic (phloem and xylem systemic) insecticide in plants, which can be used to control hidden and soil living sucking pests, such as aphids and whiteflies [3].

The carrier-mediated strategy has been used to develop novel phloem mobile insecticides [1]. Several attempts have been made to achieve phloem-mobile insecticides by introducing a carboxyl group, amino acid, or sugar to the parent compounds of existing non-phloem-mobile pesticides [2,4,5]. Pioneering work was conducted to achieve phloem-mobile pronematicide by introducing glucuronic acid moiety to oxamyl [4]. One vectorization strategy of agrochemicals has been established by introducing carboxyl group, amino acid, and sugar to phenylpyrrole fungicide [6,7,8]. Plant amino acid and sugar transporters have been reported to interact with pesticide conjugates and enhance their phloem translocation [9,10].

**Figure 1:**
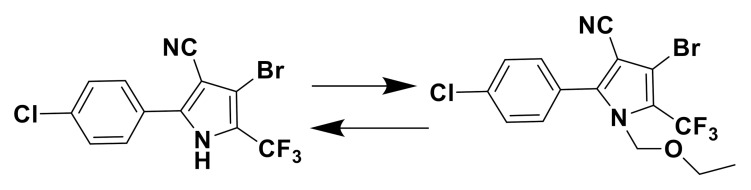
Chemical structure of tralopyril and chlorfenapyr.

Tralopyril (Figure 1) is a biocide optimized from dioxapyrrolomycin that shows excellent insecticidal activity [11,12]. However, this compound exhibits phytotoxic effects. Chlorfenapyr is the first commercial proinsecticide developed by introducing ethoxymethyl group into the pyrrole N of tralopyril [11,12,13,14]. Tralopyril is a nonsystemic insecticide without phloem mobility. To address the problems of systemicity and phytotoxicity, a strategy of vectorizing the pro-insecticide of tralopyril was proposed in our research group. The six new conjugates were designed and synthesized by introducing glucose, methyl glucuronate, and glucuronic acid moieties into tralopyril. The phloem mobility results demonstrated that our strategy can improve the phloem mobility of tralopyril [15,16].

Our previous results demonstrated that introducing glutamic acid moieties can improve the phloem mobility of tralopyril [16]. However, the structure–activity relationship of tralopyril conjugates has not yet been investigated, especially for the linker structure. In previous studies, the linker structure between the nutrient promoiety and active ingredient influence the bioactivity and phloem mobility of the conjugates [8,17]. Hence, the aim of the present study is to elucidate the structure–activity relationship for the linker between amino acid promoiety and tralopyril. (I) Nine tralopyril conjugates containing glutamic acid with different linker lengths were synthesized. (II) The insecticidal activity of the conjugates against the third-instar larvae of *P. xylostella* was evaluated using a poison fodder method. (III) Phloem mobility test in castor bean seedlings was performed. (IV) The metabolism of conjugates **42** and **43** in *P. xylostella* was investigated. This study provides a fundamental understanding of the influence of different linker lengths on the insecticidal activity and phloem mobility of new tralopyril derivatives.

## 2. Materials and Methods

### 2.1. Synthesis

#### 2.1.1. General Information for Synthesis

Solvents were of analytical grade and were dried according to the methods of the Purification of Laboratory Chemicals, Fifth Edition. Reagents were purchased from a commercial company. ^1^H NMR and ^13^C NMR spectra were obtained on a Varian INOVA-400 instrument. Chemical shifts were expressed in δ(ppm) values, with TMS as an internal standard. Analytical thin-layer chromatography (TLC) was performed on silicagel GF254. Silica gel (100–200 and 200–300 mesh, Qingdao Marine Chemical Ltd., Qingdao, China) was used for column chromatography. 

#### 2.1.2. Synthesis of Compound **4**

Synthesis of the title compound was accomplished according to a previously reported procedure [16]. NaH (60% in oil, 1 g, 25 mmol) was added to the solution of tralopyril (3.5 g, 10 mmol) in a mixture solvent of dry THF (30 mL) and bromochloromethane (12.94 g, 100 mmol, 5 equiv). The mixture was then stirred at room temperature for 2 h. Propargyl alcohol (10 mmol) in THF (10 mL) was added dropwise and stirred at 65 °C for 48 h. The reaction mixture was quenched by adding ice water, and the resultant mixture was extracted with ethyl acetate (20 mL × 3). The combined organic layers were washed with aqueous sodium hydrogen carbonate and brine, dried with sodium sulfate, filtered, and evaporated in vacuo. The residues were purified by column chromatography to obtain the desired product **4** as a white solid with 63% yield. ^1^H NMR (400 MHz, DMSO-*d*_6_) δ 7.69 (m, 4H, Ar-H), 5.38 (s, 2H), 4.05 (d, *J* = 2.4 Hz, 2H), 3.37 (t, *J* = 2.4 Hz, 1H). ^13^C NMR (100 MHz, DMSO-*d*_6_) δ 144.60, 135.88, 132.04 × 2, 129.32 × 2, 125.26, 121.31, 118.66, 113.61, 103.44, 97.96, 78.10, 77.96, 74.77, 55.65. API-ES (*m*/*z*): 441. 00 [M + Na]^+^.

#### 2.1.3. General Procedures for Compounds **14**–**22**

Synthesis of compounds **14**–**22** followed the procedure previously reported by Lu and Bittman [16,18]. In a 20 mL flask, compounds **5**–**13** (10 mmol), NaN_3_ (0.85 g, 13.2 mmol), and n-Bu_4_NBr (80 mg, 0.25 mmol) were mixed and stirred for 15 h at 110 °C. After the mixture had cooled, the product was taken up with CH_2_Cl_2_ (20 mL), and the precipitate (consisting of NaBr, unreacted NaN_3_, and phase-transfer catalyst) was removed by filtration. The salt was washed with CH_2_Cl_2_ (20 mL). Evaporation of the solvents resulted in a yellow residue that was purified by distillation at 35 °C to yield compounds **14**–**22** as a colorless liquid. 

#### 2.1.4. General Procedures for Compounds **23**–**31**

Synthesis of the title compound was accomplished according to a previously reported procedure [16]. Compounds **23**–**31** were synthesized using this procedure. Tralopyril derivative **4** (10 mmol) and crude azide **14**–**22** (10 mmol) were dissolved in 20 mL DMF. The reaction was initiated by the addition of CuSO_4_·5H_2_O (4 mmol) and sodium ascorbate (8 mmol) in 5 mL distilled water. The mixture was stirred at 60 °C until TLC indicated the disappearance of the starting materials. The mixture was poured into distilled water (20 mL), and the product was extracted three times with ethyl acetate (15 mL × 3). The organic layer was dried with Na_2_SO_4_ and filtered. The solvent was removed under reduced pressure. The residues were purified by column chromatography to obtain the compounds **23**–**31**.

Compound **23**: ^1^H NMR (400 MHz, DMSO-*d*_6_) δ 7.89 (s, 1H), 7.64~7.67 (m, 4H), 5.38 (s, 2H), 5.02 (s, 1H), 4.41 (s, 2H), 4.36 (t, 2H, *J* = 3.6 Hz), 3.74 (s, 2H). ^13^C NMR (100 MHz, DMSO-*d*_6_) δ 144.35, 141.77, 135.75, 131.80, 129.16 × 2, 125.20, 124.52 × 2, 120.77, 119.05, 113.74, 103.13, 97.82, 75.28, 61.30, 59.72, 52.02. API-ES (*m*/*z*): 528.3 [M + Na]^+^.

Compound **24**: ^1^H NMR (400 MHz, DMSO-*d*_6_) δ 7.91 (s, 1H), 7.65 (m, 4H), 5.37 (s, 2H), 5.02 (s, 1H), 4.43 (s, 2H), 4.38 (t, 2H, *J* = 4.8 Hz), 3.39 (t, 2H, *J* = 4.0 Hz), 1.90~1.95 (m, 2H). ^13^C NMR (100 MHz, DMSO-*d*_6_) δ 144.35, 141.92, 135.81, 131.79, 129.17 × 2, 125.20, 124.13 × 2, 120.78, 118.99, 113.48, 103.17, 97.87, 75.23, 61.33, 57.33, 46.57 32.88. API-ES (*m*/*z*): 542.3 [M + Na]^+^.

Compound **25**: ^1^H NMR (400 MHz, DMSO-*d*_6_) δ 7.91 (s, 1H), 7.65 (m, 4H), 5.37 (s, 2H), 5.34 (s, 1H), 4.43 (d, 2H, *J* = 4.4 Hz), 4.34~4.36 (m, 2H), 3.42 (t, 2H, *J* = 4.0 Hz), 1.80~1.83 (m, 2H), 1.34~1.38 (m, 2H). ^13^C NMR (100 MHz, DMSO-*d*_6_) δ 144.33, 142.00, 135.77, 131.78, 129.14 × 2, 125.19, 124.03 × 2, 120.75, 118.97, 113.45, 103.13, 97.85, 75.24, 61.30, 59.90, 49.20, 29.10, 26.65. API-ES (*m*/*z*): 556.3 [M + Na]^+^.

Compound **26**: ^1^H NMR (400 MHz, DMSO-*d*_6_) δ 7.90 (s, 1H), 7.62~7.65 (m, 4H), 5.37 (s, 2H), 4.48 (s, 1H), 4.44 (s, 2H), 4.30 (t, 2H, *J* = 4.8 Hz), 3.38 (t, 2H, *J* = 4.4 Hz), 1.75~1.80 (m, 2H), 1.42~1.46 (m, 2H), 1.21~1.27 (m, 2H). ^13^C NMR (100 MHz, DMSO-*d*_6_) δ 144.33, 141.89, 135.79, 131.78, 129.15 × 2, 125.19, 123.91 × 2, 120.76, 118.98, 113.46, 103.13, 97.86, 75.18, 61.32, 60.38, 49.32, 31.71, 29.58, 22.40. API-ES (*m*/*z*): 570.2 [M + Na]^+^.

Compound **27**: ^1^H NMR (400 MHz, DMSO-*d*_6_) δ 7.91 (s, 1H), 7.62~7.66 (m, 4H), 5.38 (s, 2H), 4.47 (s, 2H), 4.37 (s, 1H), 4.31 (t, 2H, *J* = 4.8 Hz), 3.39 (t, 2H, *J* = 3.6 Hz), 1.76~1.81 (m, 2H), 1.38~1.43 (m, 2H), 1.29~1.34 (m, 2H), 1.19~1.24 (m, 2H). ^13^C NMR (100 MHz, DMSO-*d*_6_) δ 144.32, 141.93, 135.87, 131.73, 129.14 × 2, 125.18, 123.89 × 2, 120.78, 118.99, 113.41, 103.11, 97.95, 75.14, 61.36, 60.60, 49.31, 32.25, 29.76, 25.69, 24.87. API-ES (*m*/*z*): 584.3 [M + Na]^+^.

Compound **28**: ^1^H NMR (400 MHz, DMSO-*d*_6_) δ 7.90 (s, 1H), 7.62~7.66 (m, 4H), 5.37 (s, 2H), 4.46 (s, 2H), 4.34 (s, 1H), 4.30 (t, 2H, *J* = 4.8 Hz), 3.38 (t, 2H, *J* = 4.0 Hz), 1.74~1.79 (m, 2H), 1.37~1.41 (m, 2H), 1.23~1.28 (m, 4H), 1.17~1.22 (m, 2H). ^13^C NMR (100 MHz, DMSO-*d*_6_) δ 144.30, 141.89, 135.81, 131.74, 129.13 × 2, 125.18, 123.88 × 2, 120.76, 118.97, 113.41, 103.11, 97.89, 75.14, 61.32, 60.64, 49.27, 32.34, 29.65, 28.23, 25.79, 25.27. API-ES (*m*/*z*): 598.3 [M + Na]^+^.

Compound **29**: ^1^H NMR (400 MHz, DMSO-*d*_6_) δ 7.91 (s, 1H), 7.62~7.66 (m, 4H), 5.36 (s, 2H), 4.46 (s, 2H), 4.33 (s, 1H), 4.30 (t, 2H, *J* = 4.4 Hz), 3.38 (t, 2H, *J* = 4.4 Hz), 1.74~1.79 (m, 2H), 1.37~1.42 (m, 2H), 1.18~1.27 (m, 8H). ^13^C NMR (100 MHz, DMSO-*d*_6_) δ 144.29, 141.89, 135.81, 131.74, 129.12 × 2, 125.18, 123.88 × 2, 120.75, 118.97, 113.39, 103.11, 97.90, 75.13, 61.32, 60.68, 49.27, 32.44, 29.67, 28.73, 28.40, 25.74, 25.37. API-ES (*m*/*z*): 612.3 [M + Na]^+^.

Compound **30**: ^1^H NMR (400 MHz, DMSO-*d*_6_) δ 7.90 (s, 1H), 7.62~7.66 (m, 4H), 5.35 (s, 2H), 4.43 (s, 2H), 4.34 (s, 1H), 4.29 (t, 2H, *J* = 4.8 Hz), 3.38 (m, 2H), 1.73~1.78 (m, 2H), 1.37~1.40 (m, 2H), 1.22~1.24 (m, 8H), 1.16~1.90 (m, 2H). ^13^C NMR (100 MHz, DMSO-*d*_6_) δ 144.29, 141.87, 135.76, 131.77, 129.13 × 2, 125.18, 123.90 × 2, 120.75, 118.96, 113.42, 103.13, 97.83, 75.15, 61.29, 60.66, 49.23, 32.45, 29.64, 29.10, 28.83, 28.28, 25.71, 25.40. API-ES (*m*/*z*): 626.5 [M + Na]^+^.

Compound **31**: ^1^H NMR (400 MHz, DMSO-*d*_6_) δ 7.90 (s, 1H), 7.63~7.66 (m, 4H), 5.34 (s, 2H), 4.42 (s, 2H), 4.47 (s, 1H), 4.29 (t, 2H, *J* = 4.8 Hz), 3.36 (t, 2H, *J* = 4.4 Hz), 1.72~1.77 (m, 2H), 1.36~1.40 (m, 2H), 1.20~1.24 (m, 10H), 1.16~1.19 (m, 2H). ^13^C NMR (100 MHz, DMSO-*d*_6_) δ 144.30, 141.87, 135.74, 131.78, 129.14 × 2, 125.18, 123.92 × 2, 120.75, 118.96, 113.45, 103.12, 97.81, 75.15, 61.28, 60.65, 49.21, 32.46, 29.62, 28.91, 28.84, 28.75, 28.28, 25.70, 25.41. API-ES (*m*/*z*): 640.2 [M + Na]^+^.

#### 2.1.5. Synthesis of Compounds **33**–**41**

Synthesis of the title compound was accomplished according to a previously reported procedure [16]. EDC (0.747 g, 3.90 mmol) and DMAP (0.293 g, 2.4 mmol) were added to a solution of Boc-Glu-OH (1.00 g, 3.90 mmol) in CH_2_Cl_2_ (5 mL), followed by **23**–**31** (3 mmol). The mixture was stirred at rt for 6 h, followed by extraction with H_2_O (3 × 5 mL) and drying with MgSO_4_. After the drying agent was filtered, the solvent was removed under reduced pressure. The residues were purified by column chromatography to obtain compounds **33**–**41**. 

Compound **33**: ^1^H NMR (400 MHz, DMSO-*d*_6_): δ 7.99 (s, 1H), 7.63~7.65 (m, 4H), 7.11 (d, 1H, *J* = 5.2 Hz), 5.38 (s, 2H), 4.62 (t, 2H, *J* = 3.6 Hz), 4.45 (s, 2H), 4.40 (t, 2H, *J* = 3.6 Hz), 3.80~3.84 (m, 1H), 2.29~2.37 (m, 2H), 1.34~1.39 (m, 20H). ^13^C NMR (100 MHz, DMSO-*d*_6_): δ 171.73, 171.18, 155.43, 144.32, 142.11, 135.81, 131.75, 129.14, 125.17, 124.51, 120.75, 119.41, 119.16, 118.96, 113.45, 103.09, 97.91, 80.29, 78.01, 75.20, 62.26, 59.62, 53.35, 48.42, 29.76, 27.99 × 2, 27.75, 27.43 × 3, 25.67. API-ES (*m*/*z*): 813.3 [M + Na]^+^.

Compound **34**: ^1^H NMR (400 MHz, DMSO-*d*_6_): δ 7.95 (s, 1H), 7.63~7.66 (m, 4H), 7.13 (d, 1H, *J* = 5.2 Hz), 5.37 (s, 2H), 4.44 (s, 2H), 4.42 (t, 2H, *J* = 4.4 Hz), 4.00~4.04 (m, 2H), 3.84~3.88 (m, 1H), 2.35~2.38 (m, 2H), 2.10~2.14 (m, 2H), 1.34~1.39 (m, 20H). ^13^C NMR (100 MHz, DMSO-*d*_6_): δ 172.00, 171.25, 155.46, 144.30, 142.00, 135.78, 131.76, 129.12, 125.17, 124.11, 120.74, 119.38, 119.13, 118.95, 113.34, 103.06, 97.88, 80.30, 78.00, 75.17, 61.27, 60.82, 53.37, 46.33, 29.84, 28.85, 27.99 × 2, 27.75, 27.46 × 3, 25.79. API-ES (*m*/*z*): 827.5 [M + Na]^+^.

Compound **35**: ^1^H NMR (400 MHz, DMSO-*d*_6_): δ 7.92 (s, 1H), 7.62~7.65 (m, 4H), 7.13 (d, 1H, *J* = 5.2 Hz), 5.36 (s, 2H), 4.44 (s, 2H), 4.35 (t, 2H, *J* = 4.4 Hz), 4.01~4.04 (m, 2H), 3.83~3.87 (m, 1H), 2.35~2.40 (m, 2H), 1.75~1.87 (m, 4H), 1.50~1.54 (m, 2H), 1.34~1.41 (m, 20H). ^13^C NMR (100 MHz, DMSO-*d*_6_): δ 172.07, 171.27, 155.44, 144.29, 141.93, 135.76, 131.76, 129.12, 125.17, 123.94, 120.74, 119.37, 119.12, 118.95, 113.35, 103.07, 97.86, 80.26, 77.98, 75.16, 63.08, 61.29, 53.36, 48.81, 29.87, 28.00, 27.76, 27.45 × 3, 26.30, 25.82, 25.03. API-ES (*m*/*z*): 841.7 [M + Na]^+^.

Compound **36**: ^1^H NMR (400 MHz, DMSO-*d*_6_): δ 7.91 (s, 1H), 7.62~7.64 (m, 4H), 7.12 (d, 1H, *J* = 5.2 Hz), 5.37 (s, 2H), 4.45 (s, 2H), 4.32 (t, 2H, *J* = 4.4 Hz), 3.98~4.04 (m, 2H), 2.34~2.39 (m, 2H), 1.79~1.82 (m, 3H), 1.58~1.60 (m, 2H), 1.35~1.39 (m, 20H), 1.24~1.29 (m, 2H). ^13^C NMR (100 MHz, DMSO-*d*_6_): δ 172.05, 171.27, 155.44, 144.27, 141.89, 135.77, 131.74, 129.11, 125.17, 123.86, 120.74, 119.40, 119.14, 118.95, 113.32, 103.06, 97.88, 80.25, 77.97, 75.13, 63.45, 61.29, 53.37, 49.04, 30.59, 29.88, 29.22, 27.99 × 2, 27.45, 27.44, 27.37, 27.31, 27.31, 25.85, 22.19. API-ES (*m*/*z*): 855.7 [M + Na]^+^.

Compound **37**: ^1^H NMR (400 MHz, DMSO-*d*_6_): δ 7.90 (s, 1H), 7.63~7.65 (m, 4H), 7.12 (d, 1H, *J* = 5.2 Hz), 5.36 (s, 2H), 4.44 (s, 2H), 4.30 (t, 2H, *J* = 4.8 Hz), 3.98~4.00 (m, 2H), 2.34~2.38 (m, 2H), 1.74~1.80 (m, 3H), 1.52~1.56 (m, 2H), 1.40~1.43 (m, 6H), 1.37~1.38 (m, 14H), 1.33~1.34 (m, 2H), 1.20~1.25 (m, 2H). ^13^C NMR (100 MHz, DMSO-*d*_6_): δ 172.06, 171.26, 155.42, 144.26, 141.85, 135.74, 131.74, 129.10, 125.16, 123.85, 120.72, 119.36, 119.11, 118.94, 113.33, 103.06, 97.84, 80.23, 77.94, 75.13, 63.59, 61.29, 53.34, 49.11, 30.59, 29.89, 29.50, 27.99, 27.82, 27.75, 27.45, 27.35, 27.32, 25.84, 25.33, 24.65. API-ES (*m*/*z*): 869.7 [M + Na]^+^.

Compound **38**: ^1^H NMR (400 MHz, DMSO-*d*_6_): δ 7.90 (s, 1H), 7.63~7.66 (m, 4H), 7.12 (d, 1H, *J* = 5.2 Hz), 5.36 (s, 2H), 4.44 (s, 2H), 4.30 (t, 2H, *J* = 4.4 Hz), 3.98~4.00 (m, 2H), 2.33~2.38 (m, 2H), 1.74~1.80 (m, 3H), 1.53~1.55 (m, 2H), 1.38~1.43 (m, 20H), 1.27~1.29 (m, 4H), 1.20~1.22 (m, 2H). ^13^C NMR (100 MHz, DMSO-*d*_6_): δ 172.06, 171.26, 155.41, 144.25, 141.85, 135.75, 131.73, 129.10, 125.16, 123.83, 120.72, 119.38, 119.13, 118.93, 113.31, 103.04, 97.86, 80.21, 77.94, 75.12, 63.68, 61.29, 53.34, 49.17, 30.59, 29.89, 29.55, 27.99, 27.94, 27.91, 27.74, 27.44, 27.34, 27.31, 25.86, 25.60, 25.10. API-ES (*m*/*z*): 883.5 [M + Na]^+^.

Compound **39**: ^1^H NMR (400 MHz, DMSO-*d*_6_): δ 7.90 (s, 1H), 7.63~7.66 (m, 4H), 7.12 (d, 1H, *J* = 5.2 Hz), 5.36 (s, 2H), 4.44 (s, 2H), 4.30 (t, 2H, *J* = 4.4 Hz), 3.98~4.00 (m, 2H), 2.33~2.38 (m, 2H), 1.75~1.78 (m, 3H), 1.53~1.55 (m, 2H), 1.41~1.43 (m, 20H), 1.26~1.28 (m, 6H), 1.18~1.21 (m, 2H). ^13^C NMR (100 MHz, DMSO-*d*_6_): δ 172.04, 171.25, 155.40, 144.25, 141.83, 135.73, 131.73, 129.09, 125.16, 123.84, 120.72, 119.36, 119.10, 118.93, 113.31, 103.03, 97.84, 80.21, 77.92, 75.12, 63.68, 61.28, 58.96, 53.33, 49.17, 30.59, 29.88, 29.59, 28.36, 28.16, 27.99, 27.74, 27.44, 27.35, 27.32, 25.85, 25.62, 25.15. API-ES (*m*/*z*): 897.5 [M + Na]^+^.

Compound **40**: ^1^H NMR (400 MHz, DMSO-*d*_6_): δ 7.90 (s, 1H), 7.62~7.64 (m, 4H), 7.11 (d, 1H, *J* = 5.2 Hz), 5.35 (s, 2H), 4.43 (s, 2H), 4.29 (t, 2H, *J* = 4.4 Hz), 3.98~4.00 (m, 2H), 2.32~2.38 (m, 2H), 1.74~1.78 (m, 3H), 1.52~1.55 (m, 2H), 1.41~1.43 (m, 12H), 1.37~1.38 (m, 14H), 1.25~1.27 (m, 4H). ^13^C NMR (100 MHz, DMSO-*d*_6_): δ 172.05, 171.26, 155.40, 144.26, 141.84, 135.72, 131.74, 129.10, 125.16, 123.85, 120.72, 119.33, 119.08, 118.93, 113.34, 103.05, 97.82, 80.22, 77.93, 75.13, 63.72, 61.27, 58.96, 53.31, 49.18, 30.59, 29.89, 29.59, 28.63, 28.45, 28.20, 27.99, 27.76, 27.46, 27.36, 27.33, 25.84, 25.66, 25.21. API-ES (*m*/*z*): 911.5 [M + Na]^+^.

Compound **41**: ^1^H NMR (400 MHz, DMSO-*d*_6_): δ 8.52 (m, 2H), 7.90 (s, 1H), 7.63~7.64 (m, 4H), 5.35 (s, 2H), 4.43 (s, 2H), 4.29 (t, 2H, *J* = 4.4 Hz), 3.97~4.00 (m, 2H), 2.32~2.38 (m, 2H), 1.73~1.78 (m, 3H), 1.52~1.55 (m, 2H), 1.41~1.43 (m, 10H), 1.37~1.38 (m, 16H), 1.24~1.26 (m, 6H). ^13^C NMR (100 MHz, DMSO-*d*_6_): δ 172.05, 171.26, 155.40, 144.26, 141.84, 135.72, 131.74, 129.10, 125.16, 123.85, 120.72, 119.33, 119.08, 118.93, 113.35, 103.05, 97.82, 80.23, 77.94, 75.13, 63.73, 61.27, 58.96, 53.32, 49.18, 30.60, 29.89, 29.61, 28.72, 28.68, 28.52, 28.26, 28.01, 27.76, 27.46, 27.37, 27.34, 25.84, 25.67, 25.24. API-ES (*m*/*z*): 925.7 [M + Na]^+^.

#### 2.1.6. Synthesis of Conjugates **42**–**50**

Synthesis of the title compound was accomplished according to a previously reported procedure [16]. Compounds **33**–**41** were added to a mixture solution of CH_2_Cl_2_ (3 mL) and TFA (3 mL). The mixture was stirred at rt for 12 h, after which the solvent was removed in vacuo. The residues were purified by column chromatography to obtain conjugates **42**–**50**.

Conjugate **42**: ^1^H NMR (400 MHz, DMSO-*d*_6_): δ 8.50 (d, 2H, *J* = 4.8 Hz), 7.96 (s, 1H), 7.61~7.63 (m, 4H), 5.36 (s, 2H), 4.36~4.41 (m, 4H), 3.98~4.01 (m, 2H), 3.75 (s, 1H), 2.42~2.50 (m, 2H), 1.95~2.10 (m, 2H). ^13^C NMR (100 MHz, DMSO-*d*_6_): δ172.32, 170.62, 144.59, 142.35, 141.98, 136.02, 131.95, 129.36, 125.29, 124.88, 120.92, 119.62, 119.37, 119.13, 113.69, 103.32, 98.03, 75.35, 62.45, 61.35, 52.28, 29.80, 28.25. API-ES (*m*/*z*): 656.2 [M + Na]^+^.

Conjugate **43**: ^1^H NMR (400 MHz, DMSO-*d*_6_): δ 8.54 (m, 2H), 7.96 (s, 1H), 7.64 (m, 4H), 5.35 (s, 2H), 4.39~4.41 (m, 4H), 3.98 (t, 2H, *J* = 3.6 Hz), 3.59 (s, 1H), 2.45~2.55 (m, 2H), 2.09 (t, 2H, *J* = 3.6 Hz), 1.91~2.02 (m, 2H). ^13^C NMR (100 MHz, DMSO-*d*_6_): δ 172.13, 170.98, 144.45, 142.13, 141.98, 135.86, 131.89, 129.26, 125.25, 124.32, 120.84, 119.44, 119.19, 119.05, 113.57, 103.24, 97.90, 75.30, 61.33, 60.97, 46.36, 29.56, 28.94, 25.73. API-ES (*m*/*z*): 670.2 [M + Na]^+^.

Conjugate **44**: ^1^H NMR (400 MHz, DMSO-*d*_6_): δ 8.53 (m, 2H), 7.92 (s, 1H), 7.63~7.65 (m, 4H), 5.35 (s, 2H), 4.35~4.40 (m, 4H), 4.02~4.04 (m, 2H), 3.61 (s, 1H), 2.40~2.45 (m, 2H), 1.97~2.01 (m, 2H), 1.51 (m, 2H), 1.40~1.43 (m, 2H). ^13^C NMR (100 MHz, DMSO-*d*_6_): δ 171.94, 168.33, 144.36, 142.08, 141.96, 135.74, 131.84, 129.59, 125.21, 124.07, 120.76, 119.30, 119.05, 118.98, 113.49, 103.17, 97.80, 75.26, 63.29, 61.27, 51.59, 28.95, 27.41, 26.30, 25.02. API-ES (*m*/*z*): 684.3 [M + Na]^+^.

Conjugate **45**: ^1^H NMR (400 MHz, DMSO-*d*_6_): δ 8.51 (m, 2H), 7.90 (s, 1H), 7.62~7.66 (m, 4H), 5.34 (s, 2H), 4.39~4.41 (m, 2H), 4.30 (t, 2H, *J* = 4.4 Hz), 3.99 (t, 2H, *J* = 4.4 Hz), 3.68 (s, 1H), 2.39~2.45 (m, 2H), 1.91~2.03 (m, 2H), 1.75~1.80 (m, 2H), 1.56~1.61 (m, 2H), 1.24~1.27 (m, 2H). ^13^C NMR (100 MHz, DMSO-*d*_6_): δ 171.96, 170.35, 144.38, 142.07, 141.96, 135.77, 131.86, 129.20, 125.23, 124.01, 120.79, 119.34, 119.08, 119.00, 113.53, 103.19, 97.81, 75.24, 63.76, 61.31, 51.91, 29.41, 29.27, 27.39, 25.53, 22.24. API-ES (*m*/*z*): 698.0 [M + Na]^+^.

Conjugate **46**: ^1^H NMR (400 MHz, DMSO-*d*_6_): δ 8.50 (m, 2H), 7.90 (s, 1H), 7.63~7.67 (m, 4H), 5.34 (s, 2H), 4.41 (m, 2H), 4.29 (t, 2H, *J* = 4.8 Hz), 3.99 (t, 2H, *J* = 4.4 Hz), 3.69 (s, 1H), 2.40~2.55 (m, 2H), 1.91~2.04 (m, 2H), 1.75~1.78 (m, 2H), 1.51~1.56 (m, 2H), 1.29~1.32 (m, 2H), 1.19~1.23 (m, 2H). ^13^C NMR (100 MHz, DMSO-*d*_6_): δ 171.92, 170.39, 144.33, 142.02, 141.89, 135.73, 131.82, 129.16, 125.20, 123.96, 120.76, 119.29, 119.04, 118.97, 113.48, 103.16, 97.78, 75.21, 63.84, 61.28, 51.89, 29.50, 29.39, 27.81, 25.53, 25.35, 24.66. API-ES (*m*/*z*): 712.5 [M + Na]^+^.

Conjugate **47**: ^1^H NMR (400 MHz, DMSO-*d*_6_): δ 8.51 (m, 2H), 7.90 (s, 1H), 7.63 (m, 4H), 5.34 (s, 2H), 4.41 (m, 2H), 4.28 (t, 2H, *J* = 4.0 Hz), 3.99 (t, 2H, *J* = 4.4 Hz), 3.74 (s, 1H), 2.44~2.52 (m, 2H), 1.94~2.02 (m, 2H), 1.73 (m, 2H), 1.53 (m, 2H), 1.27 (m, 4H), 1.18 (m, 2H). ^13^C NMR (100 MHz, DMSO-*d*_6_): δ 172.00, 170.68, 144.41, 142.07, 141.96, 135.82, 131.88, 129.23, 125.26, 124.06, 120.83, 119.41, 119.16, 119.04, 113.57, 103.23, 97.86, 75.26, 64.03, 61.34, 51.90, 29.62, 29.41, 28.17, 27.97, 25.67, 25.54, 25.17. API-ES (*m*/*z*): 726.3 [M + Na]^+^.

Conjugate **48**: ^1^H NMR (400 MHz, DMSO-*d*_6_): δ 8.50 (m, 2H), 7.90 (s, 1H), 7.64 (m, 4H), 5.34 (s, 2H), 4.41 (m, 2H), 4.28 (t, 2H, *J* = 3.6 Hz), 3.98 (t, 2H, *J* = 4.4 Hz), 3.71 (s, 1H), 2.43~2.50 (m, 2H), 1.93~2.02 (m, 2H), 1.74 (m, 2H), 1.53 (m, 2H), 1.24 (m, 6H), 1.13~1.16 (m, 2H). ^13^C NMR (100 MHz, DMSO-*d*_6_): δ 172.26, 170.33, 144.36, 142.02, 141.91, 135.78, 131.84, 129.19, 125.22, 124.01, 120.79, 119.36, 119.11, 118.90, 113.51, 103.17, 97.83, 75.22, 63.88, 61.31, 52.70, 29.70, 29.63, 28.43, 28.22, 28.03, 25.91, 25.68, 25.22. API-ES (*m*/*z*): 740.3 [M + Na]^+^.

Conjugate **49**: ^1^H NMR (400 MHz, DMSO-*d*_6_): δ 8.52 (m, 2H), 7.90 (s, 1H), 7.60~7.64 (m, 4H), 5.35 (s, 2H), 4.46 (m, 2H), 4.29 (t, 2H, *J* = 4.4 Hz), 3.97~3.99 (m, 3H), 2.35~2.41 (m, 2H), 1.87~1.96 (m, 2H), 1.74~1.76 (m, 2H), 1.50~1.56 (m, 4H), 1.40~1.42 (m, 6H), 1.18~1.19 (m, 2H). ^13^C NMR (100 MHz, DMSO-*d*_6_): δ 172.49, 171.09, 144.28, 142.02, 141.90, 135.89, 131.69, 129.13, 125.17, 123.86, 120.77, 119.58, 119.33, 118.98, 113.30, 103.04, 97.99, 75.10, 63.75, 61.35, 50.67, 29.93, 29.71, 28.75, 28.56, 28.32, 28.10, 27.49, 25.78, 25.35. API-ES (*m*/*z*): 754.3 [M + Na]^+^.

Conjugate **50**: ^1^H NMR (400 MHz, DMSO-*d*_6_): δ 8.54 (m, 2H), 7.91 (s, 1H), 7.62 (m, 4H), 5.35 (s, 2H), 4.43 (m, 2H), 4.28 (t, 2H, *J* = 4.4 Hz), 4.00 (t, 2H, *J* = 4.4 Hz), 3.94 (s, 1H), 2.53~2.57 (m, 1H), 2.39~2.44 (m, 1H), 2.02~2.07 (m, 2H), 1.72~1.77 (m, 2H), 1.53~1.55 (m, 2H), 1.43 (m, 8H), 1.21~1.24 (m, 2H). ^13^C NMR (100 MHz, DMSO-*d*_6_): δ 172.10, 168.19, 144.36, 141.98, 141.15, 135.87, 131.81, 129.19, 125.24, 124.02, 120.82, 119.53, 119.27, 119.03, 113.45, 103.12, 97.95, 75.19, 64.14, 61.36, 51.68, 29.71, 29.04, 28.83, 28.78, 28.62, 28.36, 28.08, 27.38, 25.78, 25.35. API-ES (*m*/*z*): 768.3 [M + Na]^+^.

### 2.2. Insecticidal Activity of Nine Conjugates against P. xylostella

Bioassay of insecticidal activity of nine conjugates against *P. xylostellaas* was described previously [15,16]. The insecticidal activity of compounds **42**–**50** and chlorfenapyragainst *P. xylostella* larvae was determined by mixing them with diet. Nine conjugates were dissolved in methanol and acetone (10/90, *v*/*v*) at three concentration levels (0.5, 1, and 2 mM). Each test solution (1 mL) and artificial diet (1 g) were placed in Petri dishes (6 cm in diameter). An artificial diet (1 g) containing methanol and acetone (1 mL, 10/90, *v*/*v*) was used as control. Ten *P. xylostella* larvae were introduced into each dish. *P. xylostella* was cultured at 26 °C, 70% relative humidity, and 16:8 h photoperiod (light/dark). The experiments were repeated three times. The mortality of *P. xylostella* was measured 24 h after culture.

### 2.3. Sap Collection from Ricinus communis L. Seedlings and Analysis

Castor bean seeds (*R. communis* L.) were obtained from the Agricultural Science Academy of Zibo, Shandong, China, and grown as previously described [19]. Six days after sowing, average-sized seedlings were selected for the experiments. Phloem sap was collected from the upper part of the hypocotyl according to previously described methods [19,20]. The endosperm of seedlings was carefully removed without bending or crushing the cotyledons. These latter organs were incubated in a buffer solution containing 20 mM MES (pH 5.0), 0.25 mM MgCl_2_, and 0.5 mM CaCl_2_ supplemented with 200 μM **42**–**45** or 200 μM chlorfenapyr. After 1 h of incubation, the hypocotyl was severed in the hook region for phloem exudation, and the collected sap was stored in ice until analysis. 

The phloem sap was analyzed by an HPLC (HP 1260 system, Agilent Technologies, Santa Clara, CA, USA, equipped with a quaternary pump, autosampler, and UV–visible detector)after dilution with methanol (phloem sap/methanol, 1/2 or 1/3, *v*/*v*). Separations were performed with a C18 reversed-phase column (5 μm, 250 × 4.6 mm i. d, Agilent Co., Santa Clara, CA, USA) at 25 °C. The mobile solvent system consisted of acetonitrile and water containing 0.1% trifluoroacetic acid (80/20, *v*/*v* for **42**–**45** and chlorfenapyr) at a flow rate of 0.5 mL/min. The detector wavelength was 205 nm for **42**–**45** and chlorfenapyr. External calibration was used to determine their concentrations. A series of standard solutions (0.5, 1, 5, 10, 25, and 50 μM) for linearities was prepared in methanol. Their linear equations were as follows: y = 7.4216x + 2.2817 (R^2^ = 0.9996) for **42**, y = 12.959x + 3.7681 (R^2^ = 0.9998) for **43**, y = 3.0498x + 1.5868 (R^2^ = 0.9922) for **44**, y = 5.1043x + 5.6425 (R^2^ = 0.9917) for **45**, and y = 30.993x + 25.683 (R^2^ = 0. 9989) for chlorfenapyr.

### 2.4. Metabolism of Conjugates **42** and **43** in P. xylostella

The metabolism of conjugates **42** and **43** in *P. xylostella* as was previously described [21,22,23]. The in vivo mechanism of compounds **42** and **43** in *P. xylostella* larvae was determined by mixing them with diet. The preparation of the artificial diet with 0.5 mM of tested conjugates was mentioned above. The experiments were repeated three times. After 24 h, the *P. xylostella* were removed, washed with normal saline, added to 0.1 mL methanol, and homogenized in an ice bath. After ultrasonic extraction for 10 min, the worms were centrifuged at 8000 r and 4 °C for 10 min. The supernatant was taken and stored at −20 °C for measurement. 

The supernatant was analyzed by LC–MS/MS. Chromatographic conditions: metal straight pipe, flow rate 0.3 mL/min; column temperature 40 °C; injection volume 3 μL; the mobile phase was B methanol 100%. Mass spectrum conditions: Pola: ESI-; acquisition time: 1 min; acquisition model: MS resolution: 45,000 (LE); acquisition rang: 50~1500 Da; collision energy 6 eV; Lockspary: 0.6 ng/mL, 10 uL/min, ESI-*m*/*z*: 554.2615, 236.1035. A series of standard solutions (0.125, 0.0625, 0.03125, 0.015625, 0.0078125 and 0.00390625 mg/L) for linearities was prepared in methanol. Tralopyril linear equation was y = 57.7547 × (R^2^ = 0.9844) (Over Zero Point).

### 2.5. Physicochemical Properties 

The physicochemical properties (molecular mass (MW), the octanol: water partition coefficient (logP), acid dissociation constant polar (pKa), number of hydrogen bond donors (HBDs), and number of hydrogen bond acceptors (HBAs)) of the conjugates **42**–**45**, tralopyril and chlorfenapyr were predicted using ACD/Percepta 14.0.0 software. 

## 3. Results and Discussion

### 3.1. Synthesis and Characterization

**Scheme 1:**
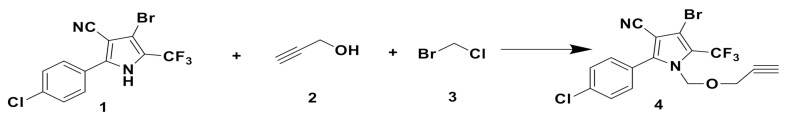
Synthetic routes of compound **4**. Reagents and conditions: THF, Bromochloromethane (5 equiv), NaH, Propargyl alcohol, 65 °C, 48 h.

**Scheme 2:**
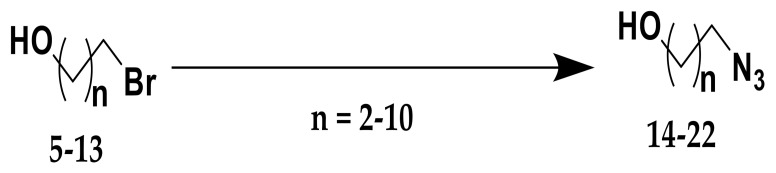
Synthetic routes of compounds **14**–**22**. Reagents and conditions: NaN_3_, n-Bu_4_NBr, 110 °C, 15 h.

**Scheme 3:**
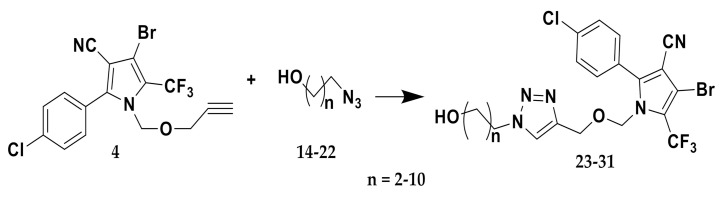
Synthetic routes of compounds **23**–**31**. Reagents and conditions: CuSO_4_·5H_2_O (0.4 equiv), sodium ascorbate (0.8 equiv), DMF, 60 °C, 3 h.

**Scheme 4:**
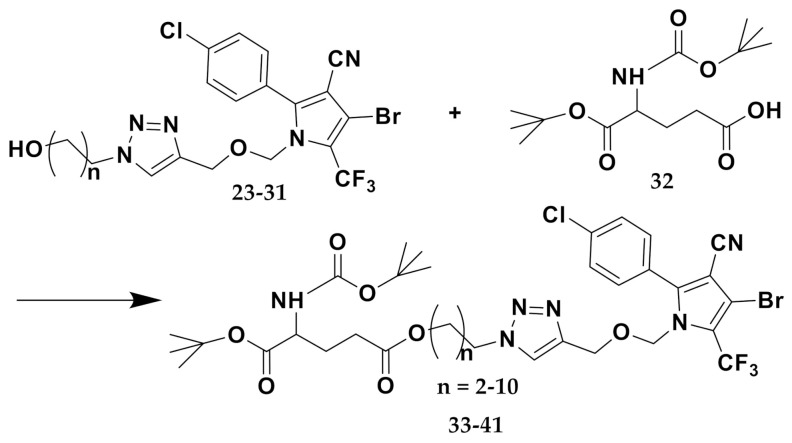
Synthetic routes of compounds **33**–**41**. Reagents and conditions: CH_2_Cl_2_, EDC, DMAP, rt, 6 h.

**Scheme 5:**
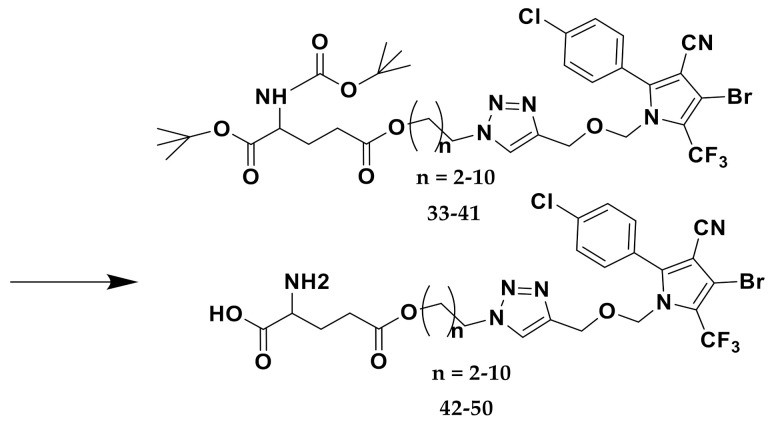
Synthetic routes of compounds **42**–**50**. Reagents and conditions: CH_2_Cl_2_, TFA, rt, 12 h.

The main conjugating method employed between glutamic acid moieties and tralopyril was click chemistry.Terminal alkyne was introduced into tralopyril to afford **4**, which reacted with azide intermediates **14**–**22** (compounds **14**–**22** were selected and prepared according to the literature) to obtain intermediates **23**–**31** via click reaction (shown in Scheme 1, Scheme 2 and Scheme 3), and the yields reached 80%. Scheme 4 shows that intermediates **23**–**31** were coupled with purchased Boc-Glu-OH to afford compounds **33**–**41** under the condition that EDC and DMAP were used together. Scheme 5 shows that the protecting groups on glutamic acidtralopyril conjugates **33**–**41** were taken off in the presence of TFA in CH_2_Cl_2_ to produce final conjugates **42**–**50** as white solids or liquid in high yields. 

The structures of compounds **4**, **23**–**31**, **33**–**41**, and **42**–**50** were confirmed by ^1^H, ^13^C NMR, and API-ES spectra. Signals of terminal alkyne could be observed in the ^1^H and ^13^C NMR spectra for compound **4**. The characteristic signals of 1-, 2-, and 3-triazole could be observed in the ^1^H and ^13^C NMR spectra of click reaction products **23**–**31**. Compounds **33**–**41** were further confirmed by the signals of the six methyl groups. The removal of six methyl groups was confirmed by the ^1^H and ^13^C NMR spectra of final conjugates **42**–**50**. The mass spectra of the 28 compounds exhibited excellent correlation with the calculated molecular mass.

### 3.2. Insecticidal Activity

The insecticidal activity of conjugates **42**–**50** against *P. xylostella* was preliminarily evaluated, and conjugates **42**, **43**, **44**, and **45** exhibited preferable activity under the tested concentrations (Table 1). According to the analysis of the linker structure–activity relationship of the nine conjugates, a longer linker chain showed a lower insecticidal activity. Based on the preliminary activity, the insecticidal activity of conjugates **42**, **43**, **44**, and **45** against *P. xylostella* was further evaluated and compared with that of chlorfenapyr over a wide range of concentrations. Table 2 shows that conjugate **42** showed the highest insecticidal activity, which may be due to the different abilities of the mixed-function oxidases to act on the conjugates of different carbon chain lengths, of which **42** was the highest. Previous studies showed that specific promoiety introduction that confers phloem mobility to pesticides usually reduced direct biological activity [2,4,5]. This phenomenon was also observed in the present work, and the 24 h LC_50_ value of these new conjugates was higher than that of tralopyril and chlorfenapyr. Hsu et al. described the strategy of phloem-mobile propesticide to overcome this inherent incompatibility [2]. Conjugates **42**–**45** shared the same O-methylene group on pyrrole N with chlorfenapyr. Thus, conjugates **42**, **43**, **44**, and **45** may have a proinsecticidal mechanism similar to that of chlorfenapyr. This inference needs to be confirmed by obtaining direct evidence in further experiments. 

### 3.3. Phloem Mobility of Conjugates

Chlorfenapyr was selected as the control in the phloem systemicity test because tralopyril has an intolerable phytotoxicity to plants. Conjugates **42**–**45** were selected for further test because of their insecticidal activity. The phloem sap of conjugates **42**–**45** or chlorfenapyr was collected within 2 h for detection. When the cotyledons were incubated in a chlorfenapyr solution at the same concentration, it was not detected in the phloem sap (Table 3). Upon observation, their concentrations in the phloem sap were approximately 24.84, 41.88, 36.77, and 30.58 μM. The detected concentrations of conjugates **43**–**45** were remarkably higher than that of conjugate **42**, and conjugate **43** had the best phloem mobility.

The physicochemical properties of conjugates **42**–**45** were calculated to predict transmembrane behavior in plants (Table 4). The “Rule of Five” can be used to predict the diffusion of endogenous molecules or xenobiotics through plant membranes [24,25]. According to this rule, poor absorption, or permeation of small molecules is more probable when their MW, logP, HBDs, and HBAs are more than 500 D, 5, 5, and 10, respectively; moreover, at least three in four parameters must satisfy this range [26]. Considering that the two parameters (MW and HBAs) of conjugates **42**–**45** in the table are out of range, they would be expected to diffuse through the membranes poorly. This result suggests the existence of a carrier-mediated absorption and transport mechanism other than passive diffusion, which is worthy of further study.

Linker length is very important for the transporter recognition of carrier-medicated conjugates [8,27]. Based on the results of the phloem systemicity test, the linker arm with the three-atom unit was the optimal length for uptake and transport of glutamic acid–tralopyril conjugates. Such an optimum in phloem mobility may be due to the structural requirement for the recognition by transporters. A shorter linker may lead to the interaction of amino acid moiety and tralopyril and affect the molecular recognition by transporters.

### 3.4. Metabolism of Conjugates ***42*** and ***43*** in P. xylostella

**Figure 2:**
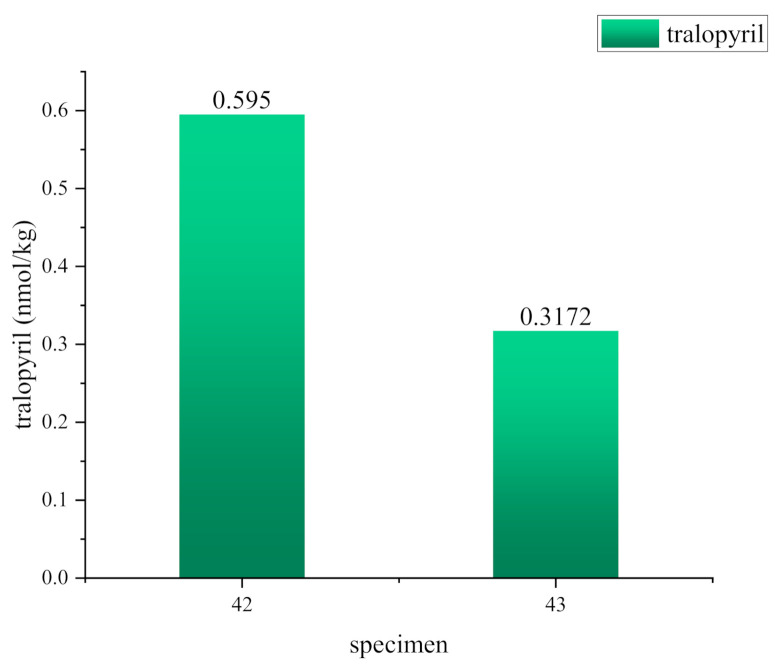
Metabolism of conjugates **42** and **43** in *P. xylostella.*

Conjugates **42** and **43** were selected to study the in vivo mechanism in *P. xylostella* and investigate the pro-insecticide properties of conjugates. Figure 2 shows that tralopyril was detected in all the *P. xylostella* treated with conjugates **42** and **43**, and the tralopyril concentrations were 0.5950 and 0.3172 nmol/kg, respectively, which is consistent with the experimental results of insecticidal activity (Table 2). The results suggest that the different linker lengths influenced the release of the active parent compound in *P. xylostella*. This behavior may involve the enzymatic bioactivation to release tralopyril because chlorfenapyr is released upon metabolism by P450s in vivo [28,29].

## 4. Conclusions

In this study, nine conjugates containing tralopyril and glutamic acid were synthesized via click chemistry with good overall yield. The results concerning insecticidal activity against *P. xylostella* indicated that conjugates **42**, **43**, **44**, and **45** exhibited preferable activity under the tested concentrations. The metabolism of conjugates **42** and **43** in *P. xylostella* showed that they can release the active compound tralopyril in vivo. The results concerning phloem mobility demonstrated that the phloem mobilities of conjugates **43**, **44**, and **45** were higher than that of conjugate **42**. Although the conjugates exhibited direct insecticidal activity that was lower than that of chlorfenapyr, conjugates **42** and **43** exhibited proinsecticide behavior. Such conjugates can achieve the primary transport of proinsecticide to the plant phloem. Then, the proinsecticide releases the active compound tralopyril at the desired site after being eaten by insects. The appropriate structural design of the linkers may enable controlling the bioactivation and phloem mobility of glutamic acid–tralopyril conjugates. This study expanded on the site-vectorized insecticide delivery strategy by investigating the effect of linker length on insecticidal activity and phloem mobility. As a promising phloem-mobile proinsecticide, the systemic insecticidal activity and phloem loading mechanism of conjugates **42** and **43** will be further investigatedin other plants.

## Figures and Tables

**Table 1:** Mortality percentage of *P. xylostella* larvae after 24 h of exposure to the diet containing the conjugates **42**–**50**.

	Concentration			
	2 mM	1 mM	0.5 mM	0.1 mM
**42**	100.00 ± 0.00 a	100.0 ± 0.00 a	100.00 ± 0.00 a	43.33 ± 0.15 b
**43**	100.00 ± 0.00 a	100.00 ± 0.00 a	76.66 ± 0.15 b	0.00 ± 0.00 c
**44**	100.00 ± 0.00 a	100.00 ± 0.00 a	73.33 ± 0.12 b	0.00 ± 0.00 c
**45**	100.00 ± 0.00 a	100.00 ± 0.00 a	60.00 ± 0.26 c	0.00 ± 0.00 c
**46**	100.00 ± 0.00 a	83.33 ± 0.12 b	48.33 ± 0.19 c	0.00 ± 0.00 c
**47**	100.00 ± 0.00 a	23.33 ± 0.12 c	3.33 ± 0.06 d	0.00 ± 0.00 c
**48**	100.00 ± 0.00 a	3.33 ± 0.058 d	0.00 ± 0.00 d	0.00 ± 0.00 c
**49**	100.00 ± 0.00 a	0.00 ± 0.00 d	0.00 ± 0.00 d	0.00 ± 0.00 c
**50**	100.00 ± 0.00 a	0.00 ± 0.00 d	0.00 ± 0.00 d	0.00 ± 0.00 c
Chlorfenapyr	100.00 ± 0.00 a	100.00 ± 0.00 a	100.00 ± 0.00 a	100.00 ± 0.00 a

Note: English letters of a, b, c and d indicate the difference in 5% level.

**Table 2:** The insecticidal activity ofconjugates **42**, **43**, **44**, and **45** against *P. xylostella.*

	LC_50_	SE	95% Confidence Limits
**42**	0.2397 mM	0.0366	0.1644 mM–0.3494 mM
**43**	0.4413 mM	0.0647	0.3311 mM–0.5882 mM
**44**	0.4400 mM	0.0624	0.3309 mM–0.5849 mM
**45**	0.4602 mM	0.0655	0.3479 mM–0.6087 mM
Chlorfenapyr	0.0249 mM	0.0145	0.0079 mM–0.0784 mM
Tralopyril	0.0224 mM	0.0601	0.0133 mM–0.0381 mM

Note: LC_50_ indicate the median lethal concentration; SE is the abbreviation of Standard error.

**Table 3:** Phloem mobility of **42**–**45** and chlorfenapyr. The cotyledons were incubated in a buffered solution containing 200 μM **42**–**45** and chlorfenapyr at pH 5.5. The sap was collected within two hour of incubation.

	Concentration in Phloem Sap (μM)
**42**	24.8493 ± 0.6730 d
**43**	41.887 ± 0.4591 a
**44**	36.7447 ± 0.6832 b
**45**	30.5837 ± 1.9420 c
chlorfenapyr	0.00 e

Note: English letters of a, b, c, d and e indicate the difference in 5% level.

**Table 4:** Predicted physicochemical descriptor MW, logP, pKa, HBD, HBA of tralopyril, chlorfenapyr, **42**, **43**, **44**, and **45** ^a^.

Feature	MW (D)	logP	pKa	HBD	HBA
Tralopyril	349.53	4.02	14 ^b^	1	2
Chlorfenapyr	407.61	4.76	14	0	3
**42**	633.81	3.00	3.91	3	11
**43**	647.83	3.22	3.92	3	11
**44**	661.86	3.52	3.92	3	11
**45**	675.88	3.76	3.92	3	11

^a^ All parameters were computed using ACD/Percepta 14.0.0 software. ^b^ Assumed nonacidic and assigned pKa of 14.
